# Handling ethical, legal and social issues in birth cohort studies involving genetic research: responses from studies in six countries

**DOI:** 10.1186/1472-6939-11-4

**Published:** 2010-03-23

**Authors:** Nola M Ries, Jane LeGrandeur, Timothy Caulfield

**Affiliations:** 1 Health Law Institute, Law Centre, University of Alberta, Edmonton, T6G 2H5, Canada; 2 Faculty of Law, University of Victoria, Victoria, Canada; 3 Bryan & Company LLP, Calgary, Canada

## Abstract

**Background:**

Research involving minors has been the subject of much ethical debate. The growing number of longitudinal, pediatric studies that involve genetic research present even more complex challenges to ensure appropriate protection of children and families as research participants. Long-term studies with a genetic component involve collection, retention and use of biological samples and personal information over many years. Cohort studies may be established to study specific conditions (e.g. autism, asthma) or may have a broad aim to research a range of factors that influence the health and development of children. Studies are increasingly intended to serve as research platforms by providing access to data and biological samples to researchers over many years.

This study examines how six birth cohort studies in North America and Europe that involve genetic research handle key ethical, legal and social (ELS) issues: recruitment, especially parental authority to include a child in research; initial parental consent and subsequent assent and/or consent from the maturing child; withdrawal; confidentiality and sample/data protection; handling sensitive information; and disclosure of results.

**Methods:**

Semi-structured telephone interviews were carried out in 2008/09 with investigators involved in six birth cohort studies in Canada, Denmark, England, France, the Netherlands and the United States. Interviewees self-identified as being knowledgeable about ELS aspects of the study. Interviews were conducted in English.

**Results:**

The studies vary in breadth of initial consent, but none adopt a blanket consent for future use of samples/data. Ethics review of new studies is a common requirement. Studies that follow children past early childhood recognise a need to seek assent/consent as the child matures. All studies limit access to identifiable data and advise participants of the right to withdraw. The clearest differences among studies concern handling of sensitive information and return of results. In all studies, signs of child abuse require reports to authorities, but this disclosure duty is not always stated in consent materials. Studies vary in whether they will return to participants results of routine tests/measures, but none inform participants about findings with unknown clinical significance.

**Conclusions:**

Analysis of how cohort studies in various jurisdictions handle key ELS issues provides informative data for comparison and contrast. Consideration of these and other examples and further scholarly exploration of ELS issues provides insight on how best to address these aspects in ways that respect the well-being of participants, especially children who become research subjects at the start of their lives.

## Background

Research with pediatric populations has generated much ethical debate [1-3]. Today, the growing number of longitudinal studies with children that involve genetic research present even more complex challenges to ensure appropriate protection of children and families as research participants [4-6]. Long-term studies with a genetic component involve collection, retention and use of biological samples and personal information over many years. Cohort studies may be established to study specific conditions (e.g. autism, asthma) or may have a broad aim to research a range of factors that influence the health and development of children. Studies are increasingly intended to serve as research platforms by providing access to data and biological samples to researchers, including those external to the cohort team, over many years.

Key ethical, legal, and social ("ELS") issues in longitudinal, genetic research studies involving children include: (1) recruitment, especially the scope of parental authority to permit a child to participate in research; (2) the nature of consent sought, particularly the breadth or specificity of initial consent, and subsequent seeking of assent and/or consent from the child; (3) confidentiality and sample/data protection measures; (4) handling sensitive information (e.g. signs of child abuse); (5) disclosure of results to participants; and (6) withdrawal from the cohort.

These issues surrounding children's participation in longitudinal research involving genetics have been the topic of much scholarly debate and analysis. Our goal in this study was to examine how a sample of birth cohort studies in North America and Europe has handled these key ELS issues. By examining their approaches, our objectives were to add to knowledge of current practices, identify similarities and differences, and determine if common practices seem to be emerging across different jurisdictions.

## Methods

We developed a questionnaire to allow for semi-structured interviews of lead investigators involved in several birth cohort studies in North America and Europe. The survey questions were informed by an extensive review of literature on children's participation in longitudinal, genetic research and selected national and international research ethics guidance documents. To provide background information for the development of our questionnaire, we also examined publicly available information about these six studies, including documents posted on the study websites or published in academic literature, such as articles that report on the study's experience in handling recruitment. The questionnaire focused on seven areas: (1) general nature of the study; (2) recruitment and enrollment; (3) consent and assent; (4) confidentiality; (5) handling of sensitive information; (6) disclosure of results; and (7) withdrawal from research. We identified approximately 20 birth cohort studies in North America and Europe through literature and online searches and selected 14 for possible inclusion in our study. Our selection was based on the objective of including studies in several countries, with either a regional or national focus, and of different sizes (from several hundred participants to 100,000 participants) and research foci to allow for comparative analysis. We also selected cohort studies that are at different stages, ranging from one that has already been underway for a decade to those that are in pilot phases.

We developed a standard invitation letter to send to lead investigators or persons identified as responsible for managing ELS aspects of the studies. The study protocol, invitation letter and questionnaire were reviewed and approved by the University of Alberta Faculties of Arts, Law and Science Research Ethics Board. We contacted lead investigators of 14 studies by e-mail to invite their participation in a telephone interview. Investigators from the following birth cohort studies agreed to participate: Born in Bradford, England ("BiB"); Canadian Healthy Infant Longitudinal Study, Canada ("CHILD"); Copenhagen Prospective Study on Asthma in Childhood, Denmark ("COPSAC"); Generation R, Netherlands ("GenR"); Growing up in France or *Etude Longitudinale Française depuis l'Enfance*, France ("ELFE"); and the National Children's Study, United States, ("NCS"). Although the invitation letters were generally sent to persons identified as a principal investigator, the interviewee was typically a member of the research team with responsibility for managing ELS aspects, such as handling ethics review applications and developing consent materials. Telephone interviews were conducted in English (by co-author JL) between September 2008 and March 2009 and average duration was 45 minutes. Interviews were audio-recorded and summary transcripts were prepared. Follow-up correspondence occurred by email to seek additional clarification or to obtain copies of documents such as consent materials, if they were available in English. Interviewees were informed they would not be individually identified in dissemination of our findings, though the cohort studies would be identified by name and policies and procedures would be attributed to specific studies. Table 1 summarises key features of the studies included in our analysis and Table 2 presents sample questions from our telephone questionnaire instrument.

**Table 1:** Participating birth cohort studies

Study	Country	Number, Start Date and Anticipated Length	Research Foci
**Born in Bradford (BIB)**	England	10,000 children Recruitment began in 2007 20 years	infant mortality, congenital anomalies and childhood disability, low birth weight, postnatal growth, childhood obesity and insulin resistance, environmental health risks (diet, water and air), renal disease, poverty, ethnicity and health, mental health

**Canadian Healthy Infant Longitudinal Development Study (CHILD)**	Canada	5,000 families Recruitment began in 2008 At least six years, likely longer	investigate the roles of indoor and outdoor environmental exposures, infections, nutrition, genetics and psychosocial factors in the development of allergic diseases, including food allergies, eczema, allergic rhinitis and asthma

**Copenhagen Prospective Study on Asthma in Childhood (COPSAC)**	Denmark	411 children Recruitment began in 1998	Clinical outcomes comprise pre-asthma, asthma, non topic dermatitis, allergic rhinitis, allergy, lung function and bronchial responsiveness

**Generation R**	Netherlands	9778 mothers and their children Recruitment began in 2001 Until adulthood	Growth, development and health from early fetal life onwards to adulthood; physical and mental outcomes, environmental and genetic outcomes

**Etude Longitudinale Française depuis l'Enfance (ELFE)**	France	20,000 Recruitment to begin in 2010 (pilot study completed) At least 20 years	Childhood development and impact of various factors (e.g. family structure, social and physical environment, education, nutrition) on physical, psychological and social outcomes to adulthood

**National Children's Study**	United States	100,000 children Recruitment began in 2009 Will follow from birth to age 21	Influence of environmental and genetic interactions on child health and development

**Table 2:** Sample questions

Topic Areas and Examples of Questions from the Telephone Survey Instrument	
Background	How many participants are included in the study? What is the focus of the research? What biological samples are collected from participants? Does the research involve visits to participants' homes?

Recruitment	How are participants recruited? Does recruitment take place at a single or multiple sites?

Consent & Assent	Describe the initial consent process with the parent(s)? Describe any process for subsequent re-consent? When, if at all, is assent sought from a child? When, if at all, is a child considered to be capable of giving their own legally valid consent?

Confidentiality	Are participant samples and records coded? How long will samples and other information be held? Who has access to samples and information?

Sensitive Information	Do you have policies/procedures in place for handling situations where researchers may have concerns that a child is suffering from abuse or neglect? Are you aware of any law in your jurisdiction that requires you to report concerns about child abuse/neglect?

Disclosure of Results	What results, if any, will be disclosed to participants? Does your study have a written policy regarding the communication of results?

Withdrawal	What are the procedures for permitting a participant to withdraw from the cohort? How are information and biological samples handled if a participant withdraws?

## Results and Discussion

All the studies we examined involve recruitment at birth or prenatally (typically at routine ultrasound appointment). Blood samples are obtained from mother, father (if available) and infant, along with other biological samples, such as breast milk, cord blood, saliva, urine and stool, with some variation among the studies in collection of these additional samples. All studies potentially involve genetic research studies within the cohort, though participants may have a choice to opt out of genetic studies and, for cost reasons, some cohort studies may not carry out genetic research will all participants. Some, such as CHILD, ELFE, GenR and NCS, involve visits to the home to collect samples of house dust or other environmental measures, or to administer questionnaires to participants. Cohort studies that focus on allergy and asthma, like CHILD and COPSAC, ask participants to undergo lung function and allergy tests. The six studies have received ethics approval and funding for a defined length of time, however, in most cases, interviewees reported that biological samples will be stored "indefinitely", or "forever". The COPSAC study has permission to hold samples until 2015, but may seek approval to retain them for a subsequent period of time. Similarly, the CHILD study plans to follow participants for six years but, with additional funding, may seek to extend the time for studying the birth cohort.

Findings in regard to each of the key ELS areas identified above are discussed here. In each section, we state how the studies we examined address the issue, then provide discussion that expands on debates and recommendations emerging from relevant literature. It is important to note that these cohort studies are subject to various levels of ethics review, which ensures that the studies' proposed handling of ELS issues are assessed in accordance with domestic ethics guidelines. Multi-site recruitment may require approval from ethics committees at each location where recruitment will occur. For example, the NCS requires approval of the National Institutes of Health institutional review board, as well as from review boards at each of the 105 study sites. The ELFE study required approval from several national bodies, including a data protection authority (Commission nationale de l'informatique et des libertés), a statistics body (Conseil national de l'information statistique), and a committee for the protection of persons (Committee du protection des personnes).

### Initial Consent

All studies require initial consent from the mother for her involvement in the research and permission for involvement of the child; paternal consent is also sought if the father is available and willing to participate in the study. The breadth of consent varies across the six studies we analysed. Potential participants in the BiB study are asked to give relatively broad consent to participate in the study as a whole, including collection and storage of biological samples for "future use" [7], but our interviewee anticipated that fresh consent would be obtained when additional information and samples are sought. Participants are advised that research partners outside the UK and Europe may have access to de-identified information and samples. Participants in the CHILD study are asked to consent to research that "will investigate some of the genetic, the immunological ..., the psychosocial and the environmental factors that may play a role in the development, the persistence and the severity of allergic disease" [8]. While the consent is relatively broad, studies must relate to allergic disease and researchers plan to re-consent participants each year during the five year duration of the cohort. COPSAC researchers must obtain ethics review approval and participant consent for new studies not addressed in the initial consent. In France, studies involving biological sampling require details on how samples will be used and stored, as well as information about future studies and whether genetic analysis will be conducted. The GenR study distinguishes between active and passive participation. Consent for active participation (*e.g*. contact with researchers for physical measures and questionnaires) is sought at four intervals: prenatal; birth to 4 years; 4 to 16 years; and > 16 years. Consent for long-term use of data (passive participation) is also sought at each phase. The NCS has a multi-stage consent process. Women who are eligible to participate are asked for their consent to participate in the study as a whole and to permit their child to participate. If a woman agrees to participate, she subsequently receives information about collection of biological and environmental samples to allow a separate consent for those aspects of the study. A woman may consent to participate only in questionnaire instruments and can refuse to provide physical samples. Participants who agree to provide samples are given a choice to opt out of genetic analysis.

While participation of a child in a birth cohort study depends on parental (or guardian) authorisation, the ethical and legal scope of a parent's role in permitting a child to become a research subject remains controversial [4]. As an NCS document points out: "A critical factor in mounting such a broad-based research effort hinges on how individuals will give permission for their children's participation....." [9]. Some ethics guidelines restrict parents' ability to permit a child's participation to studies involving no more than minimal risk [10,11]. There is, however, no consensus on the level of risk involved in birth cohort studies involving genetic research [4,12,13], though some studies self-define the risks involved as minimal [9]. We discuss below our position that children should have opportunities as they mature to confirm or reject parents' decisions to permit research participation.

Another major debate in long-term studies involving biobanking centers on the ethical and legal acceptability of broad or open consent [14-16], which refers to consent for unspecified future uses of information and samples, opposed to specific, informed consent for each use. Some ethics guidelines stipulate that broad consent should be used only exceptionally. For example, a 2008 UNESCO report on consent states: "It is not acceptable to ask a participant in a research project to give an *overall prior consent *(so-called 'blank consent') to the effect that they would agree to any study that can be carried out with the data/material they provided, unless the data/material be irretrievably unlinked to the participants" [[17], p. 24, emphasis in original]. Some study documents we examined note the challenges of informed consent in a long-term study, including the fact that all future uses of the information and samples cannot be predicted at the time of initial collection. For example, the BiB protocol states that "... samples should be processed in such a way as to enable the widest possible range of analytical tests to be conducted. This is in recognition of the fact that it is not possible to predict from the outset the scope of specific analyses" [[7], p. 24].

The ways in which the studies we examined are handling consent reveals how they are navigating the complexities of long-term studies where all future uses of information and samples cannot be predicted. Notably, none of the studies adopts a blanket approach to consent where participants are asked to give one-time consent to unlimited future use. Rather, as all studies involve ongoing contact with participants, opportunities are available to refresh the consent given at the time of original enrollment and obtain consent for specific activities (such as home visits, blood draws and physical measures like allergy testing) where particular risks can be discussed. Continuing contact with participants also provides opportunities for them to ask new questions and to exercise their right to withdraw. Recognizing that participants may have special concerns about genetic research, some cohort studies also have specific consent processes to enroll cohort members in studies involving DNA analysis. A requirement to obtain ethics review for new research outside the original scope is also common across these cohort studies.

### Assent and Consent from the Child

The NCS has established a policy that assent will be sought from children when they are between ages seven and eight. Upon reaching the age of legal majority in their state of residence, participants will go through a full informed consent process. Because the CHILD study will follow children only to age five, the protocol does not address the issue of assent or consent, though in our interviewee's experience, assent is typically sought around age seven to eight in longitudinal studies with children. At the time of our interview, the BiB study had not established an assent policy, but state they will need to address assent and consent from children as they mature. Our interviewee stated that the law in the UK regarding minors and consent to research is unclear, though, in practice, adolescents are often asked for consent around age 16. Continuing permission from parent(s) would also likely be sought until the child reaches the legal age of majority. Likewise, the ELFE study has not yet decided this issue, but anticipates seeking assent around age ten to 12 and will seek further guidance from ethics committees about obtaining consent from minors as they mature. The COPSAC and GenR studies have established policies about obtaining consent from participants in adolescence. Consent will be sought from COPSAC participants between age 15 and 17; in GenR, consent will be sought at age 16.

A large body of literature articulates the ethical and legal imperatives for seeking assent and consent from children [18-20]. Birth cohort studies, in which children become research subjects at or before birth, raise special consent considerations. By the time young subjects reach an age where they are able to understand information about the cohort study and to express views about participating, biological samples and much information about them have already been collected and used for research. Also, while " [c]hildren are a vulnerable research population, in the sense that they lack the capacity for consenting to their participation ... children's vulnerability is temporary and does not arise from a disorder; most children will become healthy adult members of society." [21] Having an opportunity to affirm or reject a parental decision made on the child's behalf is critical to respect the developing maturity of the child and their interests in making autonomous choices.

Some ethics guidelines and legal rules require assent from minors [4,11]. Children typically develop some ability to understand information about research and express preferences by age seven; by early adolescence, some young people have capacity comparable to an adult to make informed choices about research participation [1,18]. Some jurisdictions recognise a "mature minor" principle that permits a minor to make legally autonomous decisions if they have sufficient capacity to understand the nature and consequences of their decision [4]. Very long-term studies, such as those that follow a child for decades, will need to seek consent from participants as they attain the age of majority prescribed by local law, commonly in the late teenage years.

By the time children can make their own choices about research participation, extensive amounts of information about them will have been collected and used for research. This fact presents complex questions investigators will need to consider. As data- and biobanks maintained by birth cohort studies are valuable resources for other researchers, to what extent should investigators responsible for the study share information and samples about children with external researchers who do not have an institutional affiliation with the various universities and other organizations involved in the study? Some scholars have recently argued that researchers who collect and store children's DNA for general population biobanks, that is, not disease specific biobanks, should "not make these DNA samples (or individual genetic sequence data) accessible outside the biobank until donors are recontacted as adults and given their own informed consent" [[21], p. 819]. Others contend that such a policy would be too restrictive, delay advancement of research on children's health, and that privacy concerns could be allayed by robust standards for data protection and research ethics oversight [22,23]. As children mature, an additional issue is whether they should they have a right to access information about themselves, such as results of questionnaires asking the parent to provide information about the child's health, development and behavior as an infant or toddler. Access to such information may have privacy implications for the parent. These types of questions have not been uniformly addressed in the studies we examined and are topics for further analysis.

### Withdrawal

The right to withdraw from research is a fundamental right [24,25] and the studies we examined were consistent in informing participants of their right to withdraw without explanation or adverse consequences. Recognizing the research value of data and samples collected over many years, the studies offer participants withdrawal options: (1) withdraw from further contact, but allow ongoing use of previously collected data and samples and continued record linkage (if the study links to other sources of data, such as health records); (2) the same as (1), but with no permission for continued record linkage; and (3) withdrawal and no permission for further use. In the latter situation, data and samples would be removed from the study, with the exception that any data/samples that have already been used and analysed cannot be withdrawn. Permission for continuing use of samples and data may be conditional on anonymisation to prevent re-identification of withdrawn participants. In addition to advising participants of the right to withdraw, the studies we examined also permit cohort members to choose not to participate in specific data or sample collection activities.

### Protection of Samples and Data

All the studies we examined emphasise strong data protection measures to reduce risks of informational privacy breaches. Samples and personal information about participants are coded with an identification number and access to participant names and contact details is restricted to a small number of key personnel. ELFE has particularly stringent procedures to protect confidentiality of participant data. Separate files with unique identifiers are created for each recorded measure about an individual. For example, a mother's maternity survey will have a different identification code than the survey she completes at two months post-partum. A third party holds the key that permits linkage of the separate files, but does not have access to the file contents. Researchers who receive the file contents do not know the specific identity of the participant. As more information about individual participants is compiled over time, additional procedures may be implemented to guard against risks of re-identification that may arise from access to a longitudinal file that contains many indirectly identifying details. For example, if researchers need to know a participants' place of residence in France, they will receive that information, but not other information, such as age and profession, that could potentially lead to re-identification.

Some studies have established special committees to advise on data handling issues, including requests for access by third parties. For instance, the NCS has a Data Access and Confidentiality Committee http://www.nationalchildrensstudy.gov/about/organization/dacc/Pages/default.aspx to establish policies on access to NCS data and a Sample Oversight Group http://www.nationalchildrensstudy.gov/about/organization/oversightgroup/Pages/default.aspx to review studies proposing to use biological and/or environmental samples. Researchers who are not directly involved in the cohort studies we examined can generally apply for access to samples and data or to propose an add-on study. Such requests will be reviewed for scientific merit and the necessity of accessing cohort materials to answer the research questions. Other oversight committees have also been established for some studies to provide guidance on ethical and scientific issues. The ELFE study has a scientific council to advise on study design issues and an ethics committee comprised of experts external to ELFE to provide guidance on ethical issues that may arise.

Policies regarding data access, particularly by third parties external to the cohort study, should be established in advance of starting recruitment into the study. To give informed consent, potential participants require details about how information and samples will be handled, such as de-identification measures, access to identifying information, and access to datasets and samples by third parties, especially those outside the jurisdiction who may not be subject to domestic law governing personal information and biological materials.

A growing body of literature examines risks of re-identification of research participants, particularly through linkages of ostensibly de-identified databases and analyses of genomic data [15,26,27]. Reports that publicly available data from genome-wide association studies can lead to individual re-identification has prompted major funders such as the National Institutes of Health and the Wellcome Trust to tighten their policies on publication of genetic data [28]. A recent legal analysis argues forcefully that the "surprising failure of anonymization" should "trigger a sea change" in laws governing collection, use and disclosure of information and legislators should impose restrictions such as limits on size and public release of databases [29]. While there continues to be debate about the degree of effort and cost required to re-identify specific individuals from de-identified data and samples, those responsible for the repositories of information and samples compiled, linked and used in cohort studies must be alert to changing standards and best practices for data protection and safeguard appropriately the privacy interests of participants. This is particularly important where researchers seek permission from participants for broad future uses of data and samples. Such requests for future use are generally accompanied by assurances of confidentiality and robust data protection; those promises must be kept to maintain public trust.

### Dealing with Sensitive Information

Research involving direct contact with children and families, especially in home visits, presents the possibility of encountering situations of suspected child abuse/neglect or harms concerning other household members. Researchers may have legal obligations to report information of abuse or neglect to authorities. Four interviewees stated they were aware of specific laws in their jurisdiction that mandate reporting of abuse/neglect; two did not identify a legal requirement but noted that reporting to social service authorities or referral to a pediatrician would occur. Two studies reported that they do not have written policies on handling this type of sensitive information, while three studies stated that the legal obligation to report to authorities is mentioned in the consent form.

One interviewee distinguished between making direct observations in home or study centre visits about evidence of harm to a child and acquiring information over the course of the cohort (*e.g*. from questionnaires) that potentially identifies a child who is at higher risk of developing a disorder. While the former situation raises immediate obligations to notify child protection officials, the latter situation relates to long-term handling of results (addressed below).

Interviewees were asked if they have policies for handling situations where a research participant other than the child is experiencing harm, such as a new mother showing signs of post-partum depression or spousal abuse. Some questionnaires ask about a new mother's level of anxiety or depression, including, in some cases, questions as direct as whether the new mother "had thoughts about committing suicide or hurting myself" or "ending it all" [30]. Interviewees stated that situations would be addressed on a case-by-case basis, with possible referral to a health care professional or report to authorities. Two interviewees stated that this issue was addressed in the consent documents.

As it can be anticipated that research with family units has the potential to reveal sensitive information, investigators should develop policies on handling suspected cases of neglect or abuse consistent with local law and should inform participants at the time of initial consent about legally mandated reporting duties. Policies should also address situations involving imminent risks of serious harm and explain to participants how such circumstances will be handled.

### Handling of Results

All the studies we examined will publish non-identifiable results in scientific journals and will disseminate findings through media such as newspapers, television and radio. Some studies organise periodic information sessions for participating families or distribute newsletters to share updates about general research findings. Studies distinguish clinically relevant information from research results where clinical significance is not yet understood and none of the studies return the latter. Practices vary in regard to return of other results. In COPSAC, for instance, participants receive results of routine measures carried out at annual visits, such as physical measures (height and weight) and results of allergy tests. Similarly, in GenR, results from tests with known clinical relevance are provided to participants and their physicians; this provision of information is considered a recruitment incentive. In the CHILD study, results of procedures such as lung inflammation and breathing tests will be provided to parents. They are also advised they "will be informed of research findings in the event of the discovery of abnormal treatable test results." [8] The NCS policy is that individual results will only be returned if they are immediately available at the time of the participant's visit to a study centre. In effect, this limits disclosure to routine data such as blood pressure, height and weight. Our interviewee noted that research analyses of samples may only be conducted months or years after collection, which creates challenges in reporting of results. The BiB study will not return results to individuals.

A review of national and international ethics guidelines indicates broad agreement that results ought to be returned to research participants if they "meet the requirements of scientific validity, clinical significance, [and] benefit" [31], such as prevention or treatment measures. The extent to which participants should be able to request results of unknown significance is an open question. Some scholars contend that participants have a privacy-based right to choose not to know information about themselves, including genetic information [31], and various ethics guidelines state that researchers ought to respect individual choices not to receive results [25,32]. A parent who gives initial consent for their child's participation in a cohort study will make decisions where researchers offer choices about receiving results, including results about the child. While many parents may wish to receive clinically relevant information about a child, some may not [33].

Cohort studies would benefit from comprehensive policies that address various categories of findings, including those with clinical significance, those of unknown significance, those with implications for biological relatives, and incidental findings [34]. Potential participants should be informed during consent discussions of policies for handling results and, where researchers plan to offer to return results, participants should have an option to choose whether to receive information and whether disclosed results will be shared with their health care providers. The evolving maturity of child participants raises additional complexities in determining how to handle results. The choices made by researchers and parents at the outset and during early years of the study will inevitably have consequences for the children. For example, lead investigators who design the study make early decisions about what findings, if any, will be offered to parents. In turn, parental decisions will impact the interests of child participants by choosing to receive or not receive results and, if results are obtained, in making choices about the child's health care or other areas of the child's life in response to the results. As children mature and researchers provide opportunities for them to give assent/consent to continuing participation, discussion with the child should address handling of results and researchers ought to consider how to give the child options for receiving information about themselves, including information accrued in infancy and early childhood.

## Conclusions

This review of six birth cohort studies in North America and Europe reveals several key commonalities and differences in handing of major ELS issues. The studies vary in how broad initial consent is expressed, but as they involve ongoing contact with participants, opportunities exist to refresh consent and to obtain new consent for activities not contemplated at the outset of the cohort. Ethics review of new studies using data and samples from cohort participants is also a common requirement. Ensuring that consent is as specific as possible and regularly renewed will help allay concern that broad approaches to consent in longitudinal research do not meet ethical and legal standards of informed consent.

The studies that will follow children past early childhood and into the adolescent years recognise a need to consider the wishes of the maturing child and to respect autonomous choices of participants, especially when they attain age of legal majority. Some studies have not yet developed policies on assent and consent from minors; when they do, it will be important to consider whether retroactive consent will be sought to permit continuing use of previously collected samples and data [21,35]. As well, investigators ought to consider whether maturing participants will have a right to access information about themselves gathered through research instruments.

All studies advise participants of their right to withdraw from research. Offering withdrawal options to participants allows those who wish only to withdraw from future contact to authorise continuing use of previously collected materials; others may withdraw fully. The former approach is a general form of consent but ethics review of future studies and possible anonymisation of data offer some protection of participants who withdraw but authorise continued use. All the studies limit access to identifiable data of participants. With increasing technological capacity to identify individuals through combinations of de-identified data sources, cohort studies will need to ensure that data protection and access policies guard against risks of re-identification.

The clearest differences among studies emerged in handling of sensitive information and return of results. All interviewees stated that signs of abuse or neglect would trigger reporting to authorities, but not all studies inform potential participants of this disclosure duty in consent materials. Likewise, not all cohort studies advise participants of procedures for handling serious risks, such as revelation of suicidal thoughts. These aspects should be explained to ensure potential participants are aware of how sensitive issues concerning health and welfare of themselves and their families will be handled. In regard to reporting of results, none of the studies intend to report findings of unknown clinical significance but vary in whether results of more routine tests and measures will be returned. Having regard to participants' informational autonomy, it is useful to offer choices about return of clinically meaningful results and to explain at the study outset how other types of results, such as incidental findings, will be handled.

Finally, some studies have established ethical and other advisory committees to provide expert guidance throughout the duration of the cohort, including advice from persons external to the study. Such committees will help ensure impartial and specialised advice, particularly in areas where studies do not yet have policies or where existing policies require amendment to reflect new circumstances. For instance, policies on return of results may need to be changed as clinical significance becomes apparent.

Birth cohort studies are complex undertakings, especially if they involve collection and storage of biological samples for genetic analyses. The complexities include logistical challenges with recruiting and carrying out research with thousands of participants over a course of years. Profound ethical, legal and social issues also exist. Analysis of how cohort studies in various jurisdictions handle key ELS issues provides informative data for comparison and contrast. Consideration of these and other examples and further scholarly exploration of ELS issues provides insight on how best to address these aspects in ways that respect the well-being of participants, especially children who become research subjects at the start of their lives.

## Competing interests

The authors declare that they have no competing interests.

## Authors' contributions

TC and NR conceived of the design of the study. NR and JL designed the questionnaire and JL conducted interviews and collated results. All author reviewed results, NR drafted the manuscript and all authors read, edited and approved the final manuscript.

## Pre-publication history

The pre-publication history for this paper can be accessed here:

http://www.biomedcentral.com/1472-6939/11/4/prepub
